# Sex‐ and skeletal muscle‐specific gene expression response of histone 3 lysine 27‐modifying enzymes by voluntary running in mice

**DOI:** 10.14814/phy2.70853

**Published:** 2026-03-29

**Authors:** Daniel Gamu, Makenna Cameron, Yasmeen Jalil, Imen Zouaoui, Jada Sangha, Jonathan Kim

**Affiliations:** ^1^ School of Kinesiology University of British Columbia Vancouver British Columbia Canada; ^2^ Department of Medical Genetics University of British Columbia Vancouver British Columbia Canada

**Keywords:** exercise, histone acetylation/methylation, muscle adaptation, skeletal muscle

## Abstract

Skeletal muscle is highly plastic and capable of remodeling its contractile and metabolic properties depending on physical demands. Such remodeling requires modification of chromatin structure to support transcriptional activation and repression of gene programs. Chromatin dynamics depend, in part, on the acetylation and methylation of histone 3 lysine 27 (H3K27), which is controlled by several enzymes that add and remove these histone marks. Several histone post‐translational modifications in muscle have been shown to be modulated by exercise. Here, we sought to examine whether major H3K27 regulators themselves are altered by endurance training. Male and female C57BL/6J mice were provided with voluntary running wheels for 6 weeks and compared to sex‐matched sedentary controls with locked running wheels. We found that exercise altered gene expression of epigenetic machinery responsible for regulating acetylation and methylation enrichment in both a muscle‐ and sex‐specific manner, including major H3K27 acetyltransferases and core components of the polycomb repressive complex‐2. Our findings add to a growing body of evidence implicating H3K27 post‐translational modifications, and thereby chromatin dynamics, as a mechanistic component of exercise‐induced muscle remodeling.

## 1 INTRODUCTION

Engaging in regular physical activity reduces the risk of many diseases, including various cancers, cardiovascular disease, and diabetes (Kyu et al., 2016). The pleiotropic benefits of exercise underlie its utility both as a primary prevention tool and to manage disease. Benefits of exercise training are seen throughout the body, including (but not limited to) the central nervous and cardiovascular systems, adipose tissues, visceral organs, and most notably skeletal muscle (Ashcroft et al., 2024). Given that skeletal muscles comprise nearly half of adult body mass (Löffler et al., 2021), maintenance of muscle health is vital to overall metabolic health and physical functioning.

Skeletal muscle can remodel various aspects of its biology in a stimulus‐dependent manner. For example, endurance exercise training can enhance mitochondrial biogenesis and lipid oxidation, antioxidant buffering capacity, and increase the content of glucose transporters to improve peripheral insulin sensitivity (Ashcroft et al., 2024; Holloszy, 1967; Ren et al., 1994). Furthermore, exercise training can alter the size and metabolic characteristics of muscle fiber‐types (Gollnick et al., 1973), including altering the proportion of “slow” versus “fast” contractile isoforms (Furrer et al., 2023; Simoneau et al., 1985). Enhancements in slow/oxidative programming contribute to improved exercise performance, fatigue resistance, and systemic metabolism with training, in addition to mitigating disease risk factors. At the molecular level, the breadth of exercise‐induced structural and metabolic remodeling is mediated by various intracellular signaling cascades that decode an exercise stimulus into an appropriate cellular response. Although much progress has been made, our understanding of the molecular checkpoints governing muscle adaptations remains incomplete. While exercise‐induced muscle remodeling involves modulation of gene programs by transcriptional activators and repressors, binding of transcription factors requires physical accessibility to exercise‐responsive genes within chromatin. To this end, much less is known about how the modification of chromatin structure controls skeletal muscle plasticity.

**FIGURE 1:**
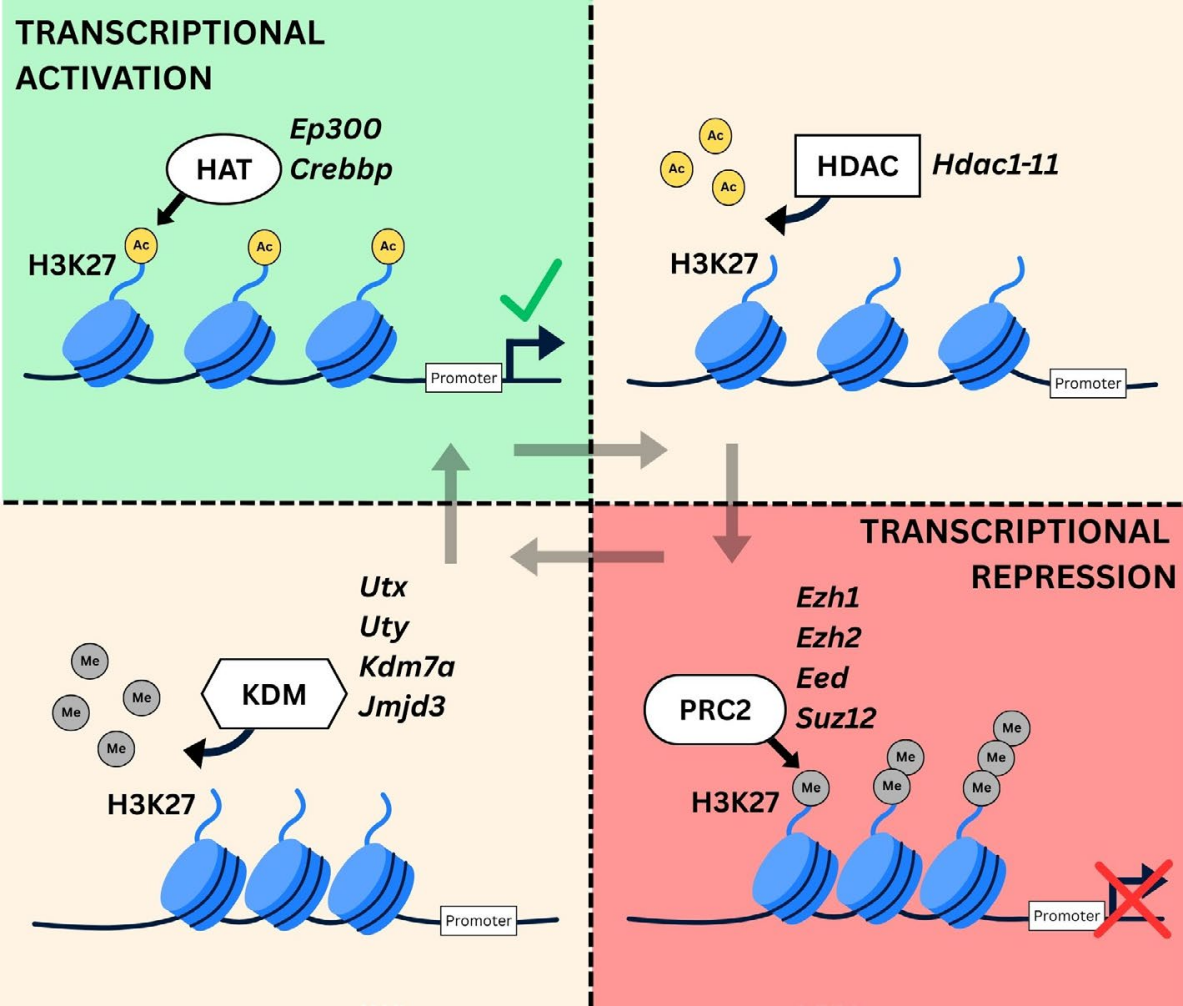
Regulation of histone 3 lysine 27 (H3K27) post‐translational modifications and gene expression. Addition of acetyl groups (Ac) onto H3K27 is a function of histone acetyltransferases (HATs), which are associated with transcriptional activation. Various histone deacetylases (HDACs) are responsible to removal of acetyl groups from H3K27. The polycomb repressive complex‐2 (PRC2) adds up to three methyl groups (Me) onto H3K27 causing chromatin condensation and transcriptional repression, while several lysine demethylases (KDMs) remove the repressive H3K27me1–3 mark.

Within chromatin, DNA is tightly wrapped around histone proteins, which assist in nuclear packaging (Gamu & Gibson, 2020). Physical accessibility of transcriptional machinery to DNA, and thereby transcription, is regulated in part by epigenetic factors, including DNA methylation and post‐translational modifications (PTMs) to histones (Gamu & Gibson, 2020). Histone (H) octamers are made of two copies each of H2A, H2B, H3, and H4 proteins. Among their numerous amino acid residues, histone 3 lysine 27 (H3K27) is unique because it is subject to two mutually exclusive PTMs with opposing effects on gene transcription (Gamu & Gibson, 2020). Acetylation (H3K27ac) facilitates chromatin “relaxation” by neutralizing positively charged N‐terminal lysine residues to destabilize DNA‐histone and histone‐histone interactions, allowing DNA binding of transcriptional machinery and transcriptional activation (Kalkhoven, 2004; Roth et al., 2001). Conversely, addition of up to 3 methyl groups (H3K27me1–3) results in tighter chromatin packaging and transcriptional silencing (Schuettengruber et al., 2017). The addition and removal of acetyl/methyl marks is a complex function of several histone acetyl/methyltransferases and histone deacetyl/demethylases, respectively (Figure 1). Acetylation of H3K27 is entirely dependent on the functionally homologous proteins p300 (*Ep300*) and cAMP‐response element binding protein (CREB) binding protein (CBP; *Crebbp*) (Jin et al., 2011; Lasko et al., 2017; Michaelides et al., 2018), whereas removal of this transcriptionally activating mark is a function of various histone deacetylases (*Hdac1‐11*). The polycomb repressive complex (PRC)‐2 methylates H3K27 to suppress gene expression. PRC‐2 is comprised of the core components embryonic ectoderm development (*Eed*), suppressor of zeste (*Suz12*), and the catalytic methyltransferases enhancer of zeste 1/2 (*Ezh1/2*) (Schuettengruber et al., 2017). Lastly, removal of methyl groups from H3K27 is a function of several lysine demethylases, including the sex‐specific *Utx* and *Uty*, *Kdm7a*, and *Jmjd3* (Schuettengruber et al., 2017). While histone PTMs have been well described in the context of establishing cellular identity during development (Gamu & Gibson, 2020), their ongoing roles following organogenesis are underappreciated and unclear, particularly within highly adaptable tissues like skeletal muscle. Mounting evidence indicates that histone PTMs, and thereby chromatin dynamics, play a central role in muscle plasticity with exercise (Seaborne & Sharples, 2020).

Genome‐wide distribution of H3K27 PTMs differs among muscle fiber‐types (Bengtsen et al., 2021; Ramachandran et al., 2019); however, they are not simply static across organismal lifespan. Histone acetylation and methylation can be impacted by even a single exercise bout in both human and rodent muscle (Masuzawa et al., 2018; McGee et al., 2009; Shimizu & Kawano, 2022; Suhr et al., 2019). Moreover, chronic endurance training can markedly alter genome‐wide and gene‐specific enrichment of H3K27ac and H3K27me1–3 (Li et al., 2024; Ramachandran et al., 2019; Williams et al., 2021), likely to support exercise‐responsive gene programs. Endurance training can alter histone octamer composition and modulate expression of histone methylating enzymes (Liu et al., 2020; Ohsawa & Kawano, 2021). These findings suggest the capacity to change chromatin structure may be integral to muscle plasticity. Although factors determining acetylation and methylation of H3K27 are complex, whether or not the capacity to lay down or remove H3K27 PTMs is modulated by exercise training has yet to be examined. Here, we sought to determine if expression of H3K27ac/me1‐3 regulatory genes fluctuates with endurance training adaptations across various mouse skeletal muscles.

## 2 MATERIALS AND METHODS

### 2.1 Animals

Male and female C57BL/6J (10–16 week old) mice were used for all studies. Mice were housed at room temperature (~22°C) under a 12 h‐light/12 h‐dark cycle in the animal facility at BC Children's Hospital Research Institute (BCCHRI) and provided ad libitum access to water and standard rodent chow (Teklad‐2918). All experiments were approved by the University of British Columbia Animal Care Committee in accordance with the Canadian Council on Animal Care guidelines.

### 2.2 Voluntary wheel running (VWR)

All mice were group housed following weaning. Prior to study commencement, littermates were randomly assigned to voluntary running wheels (VWR; male *n* = 8, female *n* = 7) or remained as sedentary controls (SED; male *n* = 8, female *n* = 6). Both VWR and SED mice were singly‐housed in shoe‐box style cages (height × width × length: 16 × 24 × 44 cm) containing food and water (as above), bedding material, a shelter, and an angled running wheel (Med Associates Inc.; Fast‐Trac™ ENV‐047 low‐profile) connected to a wireless device hub (Med Associates Inc.; DIG‐807). SED controls had their wheels locked for the entire duration of the experiment. Acquisition of running wheel data was collected using Wheel Manager Software (Med Associates Inc.; SOF‐860). Running volume (km) was continuously monitored for 6 consecutive weeks, with physical activity data expressed as average daily running distance traveled within weeks 1 to 6 (i.e., km/day). Additionally, body mass (g) was measured at the beginning of each week. Week 4 activity and week 2 body mass data were missing for female mice. Following the experiment, all running wheels were locked for 48 h prior to tissue collection.

### 2.3 Body composition and tissue collection

**FIGURE 2:**
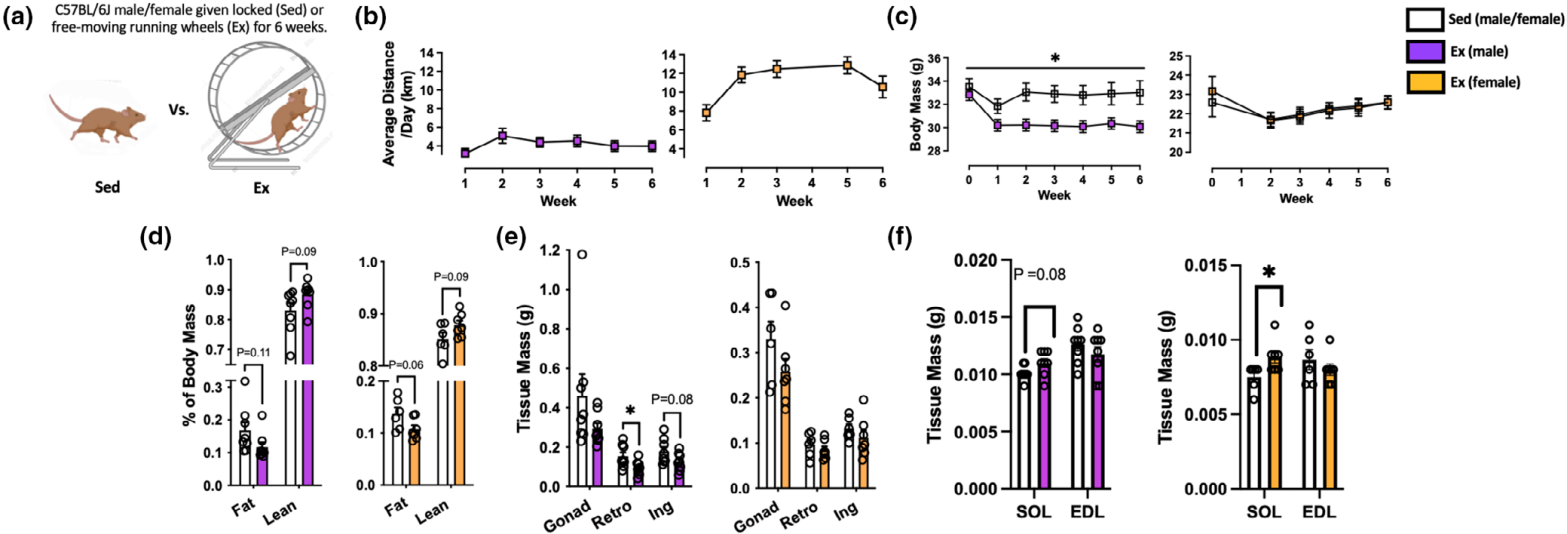
Body mass and composition response to 6 weeks of voluntary wheel running. (a) Mice were given ad libitum access to free moving (Exercise; Ex) or locked (Sedentary; Sed) running wheels for 6 weeks. (b) Average daily distance run (km) over 6 weeks. (c) Weekly body mass (g) of Sed and Ex mice. (d) Body composition (% fat/lean mass) as measured by quantitative magnetic resonance. (e) Visceral and subcutaneous adipose depot mass (g). Gonad: gonadal; Retro: retroperitoneal; Ing: inguinal. (f) Skeletal muscle mass (g). SOL: soleus; EDL: extensor digitorum longus. Values are mean ± S.E.M. *n* = 6–8/group. *p < 0.05.

Prior to (week 0) and immediately after (week 6) wheel running, body composition (i.e., lean and fat mass) was measured in non‐anesthetized mice by quantitative magnetic resonance (EchoMRI‐100H, Echo Medical Systems) as we have previously done (Gamu et al., 2023). Forty‐eight hours after wheel‐lock, mice were anesthetized with an isoflurane/O_2_ mixture and euthanized by cervical dislocation. Visceral (gonadal and retroperitoneal) and subcutaneous (inguinal) adipose depots were removed and weighed; inguinal depots were collected along the entire inguinal crease, after which lymph nodes were removed. All skeletal muscles were cleared of visible blood and connective tissue prior to weighing and snap freezing in liquid nitrogen (LN_2_). Soleus and extensor digitorum longus muscles were excised from proximal to distal tendon. Segments of red and white sections of the gastrocnemius and quadricep were partitioned by excising visually distinct portions from the muscle belly. Red/white gastrocnemius and quadricep wet weights were not presented in Figure 2 as they do not represent the entirety of each muscle. All skeletal muscles were weighed, snap frozen in LN_2_, and then stored at −80°C until analysis.

### 2.4 qPCR and Western blotting

Muscle samples were homogenized in TRIzol using a bead mill (Next Advance; Bullet Blender) and 0.5 mm zirconium oxide beads. Total RNA was column purified (RNeasy Mini Kit, Qiagen) according to manufacturer instructions, after which 0.5 μg of total RNA was reverse‐transcribed (Invitrogen; SuperScript™ III, Cat. No 18080‐051) using oligo(dT)_20_ primers. Following cDNA synthesis, samples were stored at −20°C. On the day of use, cDNA was diluted 1:10 in nuclease‐free H_2_O, followed by transcript amplification according to manufactures instructions (Promega; GoTaq®, Cat. No A6001) using a ViiA 7 PCR system (Applied Biosystems) set to Standard cycling conditions. Gene expression data was normalized to *Actb*, and calculated using the 2^−ΔΔCT^ method. Primer pairs are listed in Table 1.

**TABLE 1:** qPCR primer pairs.

Gene	H3K27 function	Forward (5′‐3′)	Reverse (5′‐3′)
Cs	–	ATGGACTAGCAAATCAGGAGG	GACACGTCTTTGCCAACTT
*Ppargc1a*	–	ATGCAGACCTAGATACCAACT	CCATCTCTCTGTCATTCCTC
*Utx*	Demethylase	ACCACAGAAATTACAGCACT	TTTGTCTCATTTGGTGTTGC
*Uty*	Demethylase	GTAGCATCTGTGAGGTGGA	TTTCGTGCACAGTTCTGACA
*Kdm7a*	Demethylase	GAAGAAGCTATTCAGGGCAT	TCTTGGCATGGTATCCAAAT
*Jmjd3*	Demethylase	GTCACTGCAGGAGGAGA	CATCAGACAGGTCGATGTTA
*Ezh2*	Methyltransferase	GGACTAGGGAGTGTTCAGT	GGCGACCAAGAGTACATTAT
*Ezh1*	Methyltransferase	AACTGTTATGCCAAAGTGGT	ATCAGCTTGGCTGTACCTA
*Eed*	PRC2 component	GTGACTATTCTTGGGCGAT	CAGTGTTGTGCATTTGGC
*Suz12*	PRC2 component	GAGCAAGAATCTCATAGCTTGTC	TGCTTTTTACCAGTAGGGAC
*Ep300*	Acetyltransferase	CATCTGGGTTTGTCTGTGA	CTCGATTCTCCAGAAAGGTC
*Crebbp*	Acetyltransferase	AGACCCTGCAGCTCTGAAAGATC	TGTCTCCCTCCACTTTCTTAGCA
*Actb*	–	TCCTTCTTGGGTATGGAATCCTG	TGGCATAGAGGTCTTTACGGA

For protein analyses, a separate portion of snap frozen muscle was homogenized in RIPA buffer supplemented with Halt protease/phosphatase inhibitor cocktail (ThermoScientific; Cat. No. 1861284) as above. Protein concentration was determined by the bicinchoninic acid assay (ThermoScientific; Cat. No. 23227) using bovine serum albumin as standards, diluted in 6X Laemmli buffer (Alfa Aeser; stock #: J61337‐AC) and boiled at 95°C for 5 min. Following gel electrophoresis, samples were wet‐transferred onto PVDF membranes (0.2 μm pore size) and probed overnight at 4°C with a cocktail of primary antibodies to detect mitochondrial respiratory chain subunits (Abcam; total OXPHOS ab110413), followed by application of an appropriate HRP‐conjugated (anti‐mouse IgG) secondary antibody (Cell Signaling Technology; 7076S). Immunoblots were digitally imaged with the Bio‐Rad ChemiDoc Imaging System following application of enhanced chemiluminescent substrates (Clarity Western ECL Substrate, BioRad; 1705061 or SuperSignal West Pico PLUS Chemiluminescent Substrate, ThermoScientific; 34579). Membranes were incubated in and normalized to Ponceau stain (BioShop; PON001), with densitometry analyses performed with ImageJ software (Version: 2.14.0/1.54f). Not all mitochondrial complexes were reliably imaged for each muscle collected; as such, only those with suitable signal were analyzed for protein expression.

### 2.5 Statistics

Mean differences between two independent groups were analyzed with a Student's two‐tailed *t*‐test, while one‐ and two‐way repeated‐measures ANOVAs followed by a Holm‐Sidak post hoc test were used where appropriate (GraphPad Prism 10.0.0). Statistical significance was determined at *p* < 0.05. All values are presented as mean ± S.E.M.

## 3 RESULTS

### 3.1 Impact of wheel running body mass, tissue weights, and mitochondrial markers

Average weekly running of males was similar to what we have previously found for C57BL/6J mice (Figure 2b) (Gamu et al., 2015). Exercise volume for females was nearly 2–3× greater than that of male mice, in line with previously documented sex differences in exercise capacity (Hastings et al., 2022). Interestingly, the body weight of exercised males was reduced by running, whereas this was not the case for females (Figure 2c). Of note, the body mass drop observed at the beginning of running wheel access may be the result of stress encountered when moving from group‐ to single‐housing conditions (Arndt et al., 2009; Hebda‐Bauer et al., 2019; Smolensky et al., 2024). Voluntary running had only a modest effect on body composition as measured by quantitative magnetic resonance (Figure 2d). While not statistically significant, fat mass tended to be lower, with lean mass correspondingly higher in exercised mice. Consistent in direction with their changes in body mass and composition, wet weights of retroperitoneal and inguinal adipose depots tended to be lower for exercised males, while no effect of exercise was seen in females (Figure 2e). Voluntary running significantly increased soleus mass in females; similar results were seen for exercised males, although this was not statistically significant (Figure 2f). No impact of exercise was seen for extensor digitorum longus mass in either sex.

**FIGURE 3:**
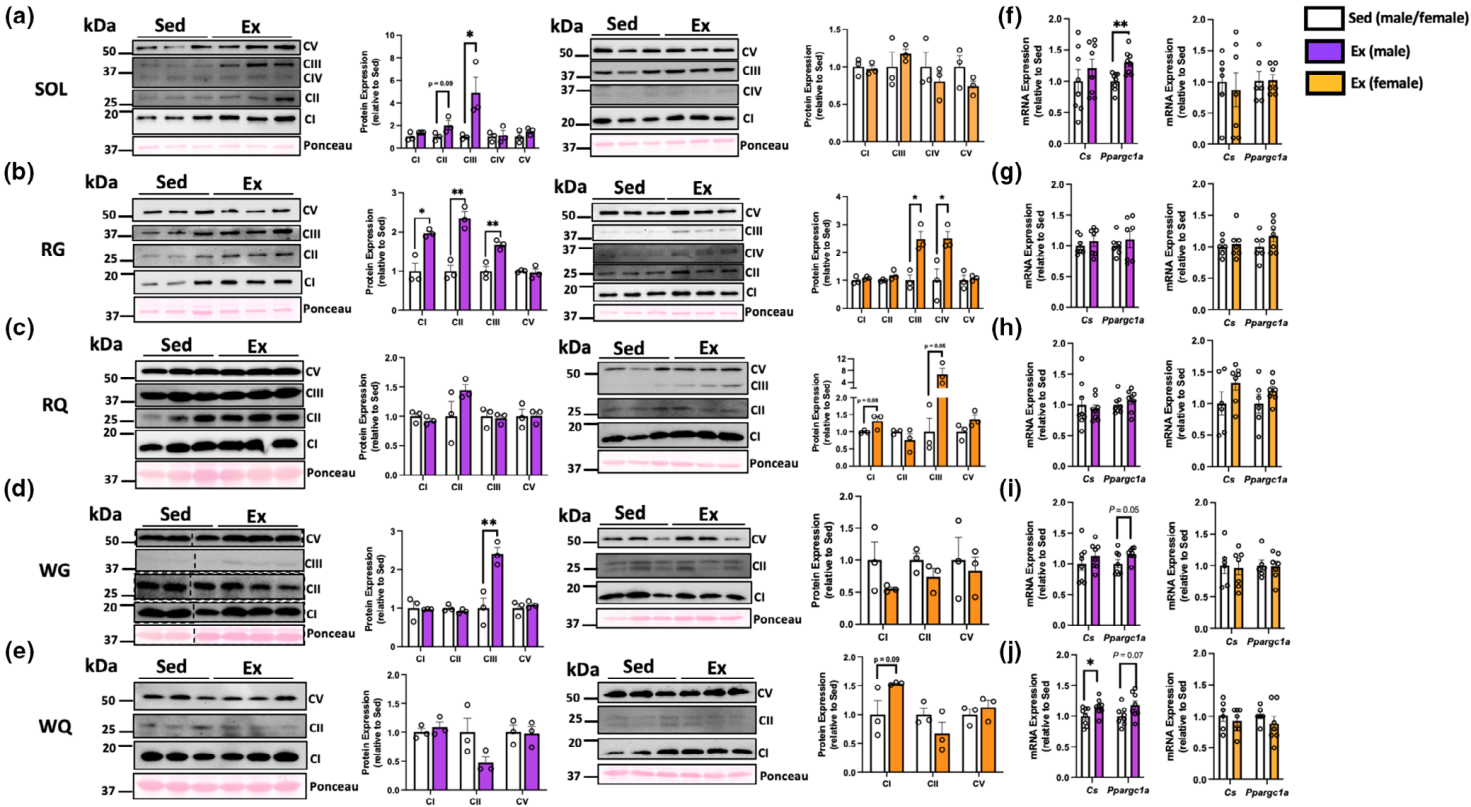
Skeletal muscle oxidative protein and gene expression following 6 weeks of voluntary wheel running. Mitochondrial respiratory chain subunit (CI‐CV) protein expression in (a) soleus (SOL), (b) red gastrocnemius (RG), (c) red quadriceps (RQ), (d) white gastrocnemius (WG), and (e) white quadriceps (WQ). Hashed lines indicate cropping of image to maintain consistent order of sample display. Protein expression (arbitrary units) from a singular Western blot was normalized to Ponceau staining and expressed relative to sedentary controls (*n* = 3/group). Oxidative gene expression within (f) SOL, (g) RG, (h) RQ, (i) WG, and (j) WQ (*n* = 6–8/group). Values are mean ± S.E.M. *p < 0.05, **p < 0.01. Ex, exercise; Sed, sedentary.

Prolonged endurance exercise enhances oxidative metabolism of muscle, in part by inducing mitochondrial biogenesis (Furrer et al., 2023). We chose to analyze an array of skeletal muscles differing in their oxidative/glycolytic capacities and fiber‐type proportions, and observed increases in mitochondrial respiratory chain subunits in a muscle‐ and sex‐specific manner. Voluntary running increased mitochondrial markers in representative slow/oxidative muscles (Figure 3a–c), with the red gastrocnemius displaying more consistent changes in male and female mice relative to sedentary controls (Figure 3b). Markers in fast/glycolytic muscles were not as robustly altered by exercise (Figure 3d/e); only complex III of male white gastrocnemius increased (Figure 3d). Wheel running increased expression of oxidative genes in a muscle‐specific manner in males only (Figure 3f–j). Expression of the mitochondrial master regulator *Ppargc1a* was significantly increased in the SOL (Figure 3f), with trending increases in WG and WQ (Figure 3d/e). Whereas *Cs* expression was significantly elevated in male WQ only (Figure 3f). Thus, the volume of voluntary running observed herein was sufficient to induce stereotypical remodeling of muscle oxidative metabolism.

### 3.2 Gene expression of H3K27‐modifying enzymes

**FIGURE 4:**
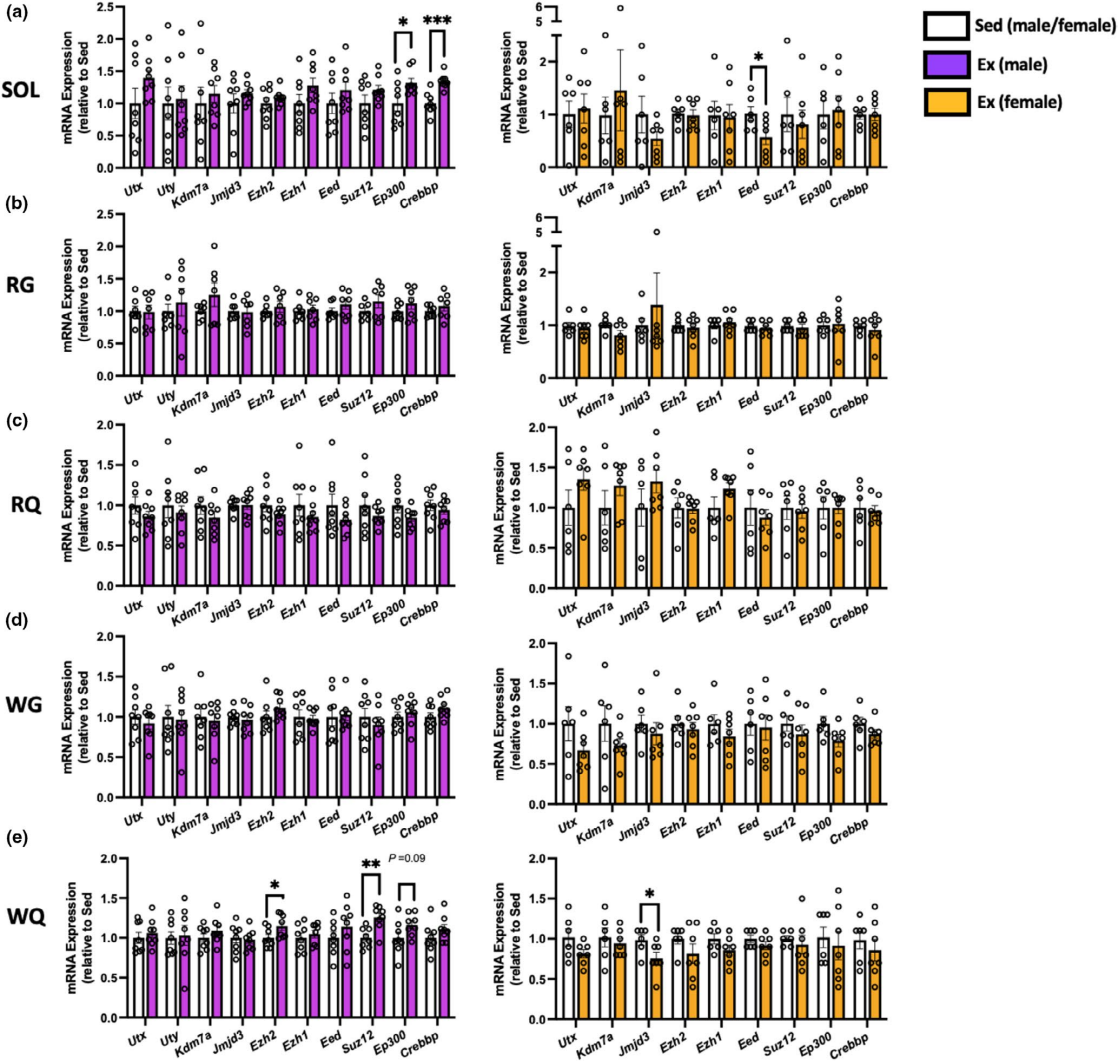
Gene expression of H3K27 demethylases, methyltransferases, and acetyltransferases to 6 weeks of voluntary wheel running in representative slow/oxidative and fast/glycolytic skeletal muscles. mRNA levels (expressed relative to Sed controls) in (a) soleus (SOL), (b) red gastrocnemius (RG), (c) red quadricep (RQ), (d) white gastrocnemius (WG), and (e) white quadricep (WQ). Values are mean ± S.E.M. *n* = 6–8/group. *p < 0.05, **p < 0.01, ***p < 0.001. Ex, exercise; Sed, sedentary.

Exercise can induce gene transcription of several “master‐regulators” of metabolic programming in muscle (Hood & Saleem, 2007), in addition to altering the enrichment of acetyl and methyl marks on H3K27 (Li et al., 2024; Lim et al., 2020; Ramachandran et al., 2019; Shimizu & Kawano, 2022). As transcriptional activation and repression are intimately linked to H3K27 post‐translational modifications, we sought to examine if gene expression of H3K27 demethylases, methyltransferases, and acetyltransferases is itself modified by physical activity. Within male SOL (Figure 4a), transcript abundance of the functionally homologous H3K27 acetyltransferases *Ep300* and *Crebbp* were both significantly upregulated following 6 weeks of wheel running, whereas no other genes were changed in this tissue. Unlike that of males, acetyltransferase gene expression was not changed by running; however, only the PRC‐2 core component *Eed* was lower in the soleus of exercised females (Figure 4a). Exercise did not impact the transcriptional response within the red gastrocnemius, red quadricep, and white gastrocnemius (Figure 4b–d), irrespective of animal sex. Several PRC‐2 genes were elevated in male white quadricep (Figure 4e), including the catalytic methyltransferase *Ezh2* and the core component *Suz12*. Although not statistically significant, *Ep300* gene expression tended to be higher following wheel running in male WQ (Figure 4e). Like that of the soleus, a sex‐specific effect of wheel running was observed in female WQ, with exercise downregulating gene expression of the H3K27 demethylase *Jmjd3* (Figure 4e).

## 4 DISCUSSION

Skeletal muscle is uniquely capable of remodeling its structure and metabolic characteristics in response to specific demands placed upon it. Such plasticity is a function of complex signaling networks that decode context‐specific information about physical activity, which involves activation and repression of gene programs. Modulating gene expression requires, among other factors, altering chromatin structure to regulate access of transcriptional machinery. Post‐translational modifications to H3K27 are well known to regulate chromatin dynamics and thereby gene expression (Figure 1). Several studies have shown that various histone post‐translational modifications and/or their regulatory enzymes are altered by exercise (Li et al., 2024; Liu et al., 2020; Ohsawa & Kawano, 2021; Ramachandran et al., 2019; Williams et al., 2021). Our objective here was to examine whether gene expression of major H3K27‐modifying enzymes specifically was responsive to physical activity. We found that 6 weeks of voluntary wheel running induced modest changes of major H3K27 acetyltransferases and PRC‐2 components in a sex‐ and muscle‐specific manner.

Voluntary wheel running is a useful paradigm of exercise training that minimizes animal stress and enables mice to run during their natural circadian pattern (Hastings et al., 2022). Weekly running volume of male mice was comparable to our previous work (Gamu et al., 2015) and others (Davidson et al., 2006; Lightfoot et al., 2004; Meek et al., 2009), although some have found higher volumes for C57BL/6J mice (De Bono et al., 2006; Lerman et al., 2002; Waters et al., 2004). In line with others (Konhilas et al., 2004), we found females to be superior runners throughout the training period. Unlike the males, female body mass was surprisingly unchanged despite running nearly 2× more than males. However, we did not measure food intake in this study, which may have increased in females to compensate for the energetic demands of running. Regardless, modest reductions in fat mass and concomitant increase in lean mass were observed in runners of each sex. Although the reduction in body fat percentage of male runners was consistent with the decline of several adipose depot wet weights, this was not the case for female runners, suggesting that other adipose depots were impacted by wheel running. The proportional increase in lean mass of running mice, regardless of sex, was consistent with the larger soleus wet weights, which has previously been shown in C57BL/6J mice following 7 weeks of weighted wheel running (Konhilas et al., 2005).

Endurance exercise training is well known to enhance skeletal muscle oxidative metabolism and increase mitochondrial biogenesis. In agreement with this, we found mitochondrial markers were increased in skeletal muscles of both sexes, indicating running volume was sufficient to elicit classical oxidative remodeling. However, we did not consistently observe increased respiratory chain protein expression across all subcomplexes and muscles in our hands, which may reflect the exercise variability inherent to voluntary running. Although oxidative proteins are common indicators of endurance training efficacy, they are by no means the only relevant adaptive readouts given the pleiotropic effect of exercise (Hawley et al., 2021). Thus, it is unlikely functional adaptations were completely absent in skeletal muscle where oxidative proteins were unchanged.

Mounting evidence indicates post‐translational modifications of histones regulate homeostatic and adaptive programming of skeletal muscle. Deacetylation of histone lysine residues is well‐known to facilitate transcriptional repression (Figure 1), and inhibiting various histone deacetylases (HDACs) in rodents can broadly recapitulate exercise adaptations, including enhancing mitochondrial biogenesis and promoting fast‐to‐slow fiber type transition (Galmozzi et al., 2013; Gao et al., 2009; Gaur et al., 2016; Potthoff et al., 2007). Exercise causes the nuclear export of HDACs in muscle (McGee et al., 2009; McGee & Hargreaves, 2004), suggesting transcriptional activation of exercise‐responsive genes is aided by HDAC subcellular redistribution. Although histone acetylation and subsequent expression of metabolic genes are enhanced by HDAC inhibition (Galmozzi et al., 2013), it remains unclear how much of this effect is driven by H3K27ac per se, particularly given that no H3K27‐specific deacetylase has been identified. Adaptive muscle remodeling via HDAC inhibition could result from greater occupancy/access to H3K27 by histone acetyltransferases. Prolonged endurance training of both humans (Williams et al., 2021) and rodents (Ramachandran et al., 2019) markedly alters genome‐wide distribution of H3K27ac, suggesting p300/CBP activity is recruited to co‐ordinate adaptive gene programming with exercise. In line with this, we provide evidence here that the capacity of p300/CBP may be enhanced in the trained state (Figure 4a), although it remains uncertain whether this translates to enhanced protein expression and/or catalytic activity in our study. Given their considerable redundancy, tissue‐specific single knockout of p300 or CBP in mice does not impact muscle homeostasis or metabolic adaptation to exercise (LaBarge et al., 2016; Martins et al., 2019), whereas inducible double knockout profoundly impairs muscle function, resulting in death (Svensson et al., 2020). As such, adaptive gene programs regulated by p300/CBP during exercise currently remain unclear.

Contrary to acetylation, methylation of H3K27 results in chromatin compaction and gene silencing (Figure 1). Gene expression of several PRC‐2 components was differentially altered between males and females by wheel running in oxidative vs. glycolytic muscles (Figure 4a/e). *Suz12* and *Eed* are each required to maintain PRC‐2 integrity (Gamu & Gibson, 2020), and knockout of either prevents H3K27me3 formation (Montgomery et al., 2007; Pasini et al., 2004). Given that *Eed* expression was lowered by running in female soleus (Figure 4a), it is possible that PRC‐2 catalytic activity may be correspondingly lower with exercise. Endurance training has been shown to reduce H3K27me3 enrichment at the *Ppargc1a* promoter (Li et al., 2024), although we did not observe changes in *Ppargc1a* and mitochondrial protein in the soleus of exercised females here. While currently unclear, exercise‐induced downregulation of *Eed* could facilitate activation of non‐mitochondrial gene programs within the soleus with exercise.

Histone methylation may serve a unique role in rodent fast/glycolytic muscles, with several studies showing enrichment of H3K27me3 with exercise (Lam et al., 2025; Ohsawa & Kawano, 2021; Shimizu & Kawano, 2022). While seemingly contrary to its canonical role in transcriptional repression, pharmacological inhibition of PRC‐2 activity impairs endurance adaptations within rodent fast/glycolytic muscle (Shimizu & Kawano, 2022), indicating active H3K27me3 deposition is required for exercise remodeling. PRC‐2 inhibition may prevent formation and maintenance of bivalent (poised) chromatin regions, which are marked by both transcriptionally repressive (i.e., H3K27me3) and activating (i.e., H3K4me3) marks. Moreover, promoting H3K4 methylation within rodent muscle mimics endurance training, including enhancing slow‐twitch programming and improved running performance (Araki et al., 2023; Liu et al., 2020). Recently, treadmill training was shown to increase plantaris EZH2 content and global H3K27me3 enrichment in C57BL/6J mice (Lam et al., 2025). In agreement with these findings and those described above, we show here that wheel running upregulated gene expression of the methyltransferase *Ezh2* along with *Suz12* in male white quad (Figure 4e). Within PRC‐2, *Suz12* helps to maintain methyltransferase activity, with knockout embryos unable to form H3K27me2/3 (Pasini et al., 2004). While unclear from our current study, wheel running may enhance the capacity of muscle to methylate H3K27 and maintain poised regions, in part by increasing PRC‐2 stability. Although wheel running did not increase *Ezh2* or *Suz12* in female white quad, gene expression of the H3K27‐demethylase *Jmjd3* was downregulated (Figure 4e). It is possible that increasing PRC‐2 or correspondingly reducing demethylase expression may aid in H3K27me3 enrichment and formation of bivalent chromatin regions previously observed in fast/glycolytic muscles (Lam et al., 2025; Ohsawa & Kawano, 2021; Shimizu & Kawano, 2022), but this requires future examination.

While we show modest gene expression changes in several regulators of H3K27 PTMs, many remained unaffected following 6 weeks of wheel running, particularly within female skeletal muscles; several reasons may account for this. Although voluntary wheel running reduces animal stress and enables long term exercise studies without a need for continuous monitoring, there is an obvious trade‐off in lack of control for exercise frequency, intensity, duration, and circadian timing of running. Secondly, although we collected tissues post‐training where we were likely to observe the greatest increase in classic mitochondrial markers, we cannot rule out that changes in H3K27 modifying genes occurred during earlier stages of the experiment. Furthermore, we did not examine genomic location, enzyme activity or subcellular distribution of H3K27‐modifers; certainly, several studies have shown endurance training modulates loci‐specific and/or global H3K27ac/me3 enrichment (Li et al., 2024; Ohsawa & Kawano, 2021; Ramachandran et al., 2019; Williams et al., 2021). Ultimately, muscle‐specific loss‐of‐function studies will be required to delineate which functional programs are regulated by individual H3K27 writers and erasers with endurance training.

## 5 CONCLUSION

Exercise improves overall health and muscle functioning by remodeling various structural and metabolic programs. This requires altering the chromatin landscape to support the activation and suppression of adaptive gene programs. Chromatin dynamics are regulated by numerous factors like post‐translational modifications to various histone residues, including H3K27. Here, we provide the first sex‐specific investigation into the impact of prolonged exercise on the gene expression of H3K27 regulators of skeletal muscle. We found that 6 weeks of voluntary wheel running altered genes responsible for both H3K27ac and me3 in a muscle‐ and sex‐specific manner. Our findings add to a growing body of evidence that histone modifications serve as a regulatory node coordinating muscle plasticity.

## AUTHOR CONTRIBUTIONS

**Daniel Gamu:** Conceptualization; data curation; formal analysis; funding acquisition; investigation; methodology; project administration; resources; supervision; visualization. **Makenna Cameron:** Data curation; formal analysis; investigation; supervision; visualization. **Yasmeen Jalil:** Data curation; formal analysis; investigation; visualization. **Imen Zouaoui:** Data curation; formal analysis; investigation; visualization. **Jada Sangha:** Data curation; formal analysis; visualization. **Jonathan Kim:** Data curation; formal analysis; visualization.

## FUNDING INFORMATION

This work was supported by an NSERC Discovery Grant (RGPIN‐2024‐06095) and Kinesiology Equipment and Research Accelerator Fund (UBC) to DG.

## CONFLICT OF INTEREST STATEMENT

The authors have none to declare.

## Data Availability

All data will be made available upon reasonable request.
